# Physiotherapy for pelvic pain and female sexual dysfunction: an untapped resource

**DOI:** 10.1007/s00192-017-3536-8

**Published:** 2018-01-09

**Authors:** Bary Berghmans

**Affiliations:** Pelvic Care Center Maastricht, Maastricht University Medical Centre, P.O.Box 5800, 6202 az Maastricht, The Netherlands

**Keywords:** Chronic pelvic pain, Sexual dysfunction, Vulvodynia, Vestibulodynia, Dyspareunia, Vaginism, Sensitization, Physiotherapy, Multidisciplinary

## Abstract

**Introduction and hypothesis:**

Chronic pelvic pain (CPP) in women is a complex syndrome. Pain sensation and intensity often do not correspond with the identified lesion location but are felt elsewhere, leading to muskuloskeletal and myofascial disorders and sexual dysfunction (SD). Although physical aspects are prevalent, they are often underdiagnosed and undertreated due to lack of understanding regarding its origin and distribution. Frequently, patients experience pelvic pain as psychological distress resulting in physical complaints, leading clinicians to prescribe medication or surgical intervention to correct or alleviate these symptoms, often with insufficient results. Because pelvic floor muscle disorders contribute significantly to CPP and SD, there is rationale for physiotherapy. However, physiotherapy is a widely underused and untapped resource, which has its place in the multidisciplinary approach to these health problems.

**Methods:**

Computer-aided and manual searches and methodological quality assessment were carried out for meta-analyses, systematic reviews, and randomized controlled trials (RCTs) published between 1990 and 2017 investigating classification, assessment, and (physiotherapeutic) treatment of pelvic pain and/or female SD defined by the keywords below. Expert opinions were sought via interviews.

**Results:**

Due to a lack of sufficient relevant medical information, referral data, and test results, focused physiotherapy is difficult to administer adequately. However, recent quality studies indicate significant clinical effects of physiotherapy for CPP and female SD, and experts advocate a multidisciplinary approach that includes physiotherapy.

**Conclusions:**

Because of its holistic approach, physiotherapy can contribute significantly to the multidisciplinary assessment and treatment of CPP and female SD.

## Introduction

Chronic pelvic pain (CPP) is defined as abdominal pain below the umbilicus for at least 6 months [1]. It is a complex and confusing health problem affecting the quality of life of many women with several types of urogynecological disorders [2], often resulting in depression, anxiety, and fatigue [3]. CPP, with its basis in the central nervous system, involves emotional, cognitive, behavioral, and sexual responses [4]. CPP syndrome (CPPS) is CPP without a proven infection or obvious local pathology and is related to symptoms suggestive of lower urinary tract, bowel, gynecological, and sexual dysfunction (SD) [3]. Pain sensation and intensity often do not correspond with the identified lesion location but are felt elsewhere, leading to a wide array of musculoskeletal and myofascial disorders and SD [4], and the syndrome frequently coexists with chronic pelvic floor dysfunction—in most cases with the presence of physical findings [5].

The underlying mechanism of this phenomena is partially explained by repeated or prolonged somatic and visceral sensory input of nociceptors, resulting in lowering their activation threshold, and sensitization of previously non-involved afferent nerve fibers. This is called peripheral sensitization [6]. Initially, functionally “silent” fibers may be activated after being sufficiently sensitized by exciting stimuli, increasing the excitability of nociceptors [7]. Electrical impulses initiate neurotransmitter release from central terminals in nociceptors that propagate the signal across synapses to dorsal-horn neurons. Greater stimulus intensities are associated with greater release of neuropeptides, including substance P, from central terminals of C fibers. This mechanism generates a greater postsynaptic response. The intense afferent bombardment of noxious information through viscerosomatic convergence, and ongoing somatosensorial input from muscle and skin at the dorsal horn of a segment in the spinal cord, leads to central sensitization perceived in the brain as prolonged, intense pain [4]. With central sensitization initiation, amplification, and perpetuation of pain, perception becomes manifest as allodynia, hyperalgesia, and referred pain.

Convergence of neural inputs often hinders precise localization and discrimination of sensory information. It also forms the basis for referred pain and explains why visceral pathologies are commonly felt as pain in somatic structures innervated by the same spinal segment [pelvic floor muscles (PFM), in particular]. Furthermore, since visceral afferent fibers terminate over several spinal segments above and below the segment level of input, referred pain may be present in areas remote from the affected visceral organ. This up-regulation of the sensory system further effects interneurons that connect to alpha and gamma motoneurons, leading to segmental overactivity of PFM, spasm, and contracture. This pelvic floor dysfunction and myofascial pain can then lead to SD, such as dyspareunia or vaginism, as the PFM tighten, becoming inflexible and incapable of accommodating penetration during intercourse.

Myofascial pain is an expression of dysfunction in the muscle and surrounding myofascial/connective tissue [7]. According to Simons et al. [8], myofascial pain has a lifetime prevalence of up to 85% in the general population. Nevertheless, physicians traditionally underdiagnose and often overlook this issue. The presence of myofascial trigger points (MTrPs) in the symptomatic region is a distinctive feature. MTrPs are small, palpable, hyperirritable nodules located on taut bands of skeletal muscle in an area of sustained contracture [9] and can be active or latent. Active points are spontaneously painful areas that do not require physical stimuli, whereas latent points are painful only upon physical palpation. Patterns of referred pain are often predictable and can be documented by anatomical mapping. MTrPs may also cause motor and autonomic disturbances, affecting the function of visceral organs [9], are commonly found in many chronic pain conditions, and, when active, typically present as a regional pain syndrome [7].

Simons et al. [10] noted that in the pelvis, MTrPs can be found in the vagina, anorectum, urethra, pubic bone, vagina, coccyx, abdomen, lower back, and backside of thighs. They may also refer pain from those areas back to the pelvic region, making myofascial pelvic pain difficult to localize [11]. Women with myofascial pelvic pain often demonstrate symptoms of dyspareunia, painful urination (dysuria), and difficulty in defecating (dyschezia), though these symptoms may be expressions of other, nonrelated, pelvic floor or pelvic viscera problems [10].

Pelvic-floor-related SD comprise vaginism, dyspareunia, and (chronic) pelvic pain. Many authors report that in patients with CPP and/or SD the role of the pelvic floor is of the utmost importance [12–14]. In 57% of women with an overactive pelvic floor, dyspareunia has been reported and is felt to be secondary to stretching of shortened PFM, stimulation of painful regions and/or local adhesions, fibrosis, or organ dysfunction [15]. Because pelvic pain following sexual activity is often sustained for up to 3 days [16], these symptoms can have significant negative impact on the integrity of physical relationships and a woman’s quality of life, inducing feelings of fear, anxiety, and depression [17]. Considering there is a clear and deep relationship between PFM disorders, CPPS, and female SD, one would expect that there is an important role for physiotherapy in these patients; in fact, however, the opposite is true. In relevant clinical practice, physiotherapy seems to be a widely underused strategy—an untapped resource—to decrease CPP and improve sexual function. In this paper, we hypothesize that physiotherapy has a place in the multidisciplinary treatment approach to women with CPPS and SD.

## Methods

To support our hypothesis, we conducted computer-aided and manual literature searches and methodological quality assessment of meta-analyses, systematic reviews, and randomized controlled trials (RCTs) published between 1990 and 2017 related to physiotherapeutic assessment and treatment of pelvic pain and/or female SD. Existing classification and models of assessment and interventions used by other relevant health care professionals were reviewed. Keywords defined were (chronic) pelvic pain, sexual dysfunction, vulvodynia, vestibulodynia, dyspareunia, vaginism, sensitization, physiotherapy, and multidisciplinary. Key-opinion leaders from gynecology, urology, sexology, and physiotherapy, all well-known experts in the field, were interviewed about their opinion and clinical expertise.

## Results

The literature search revealed 109 studies; 32 met our criteria, of which there were no meta-analyses, 27 (systematic) reviews, and five RCTs.

### Classifications and models

To more reliably diagnose female SD, we consulted the American Physiatric Association’s (APA)* Diagnostic and Statistical Manual of Mental Disorders 5* (DSM-V) of classified mental disorders with associated criteria [1]. These classifications include psychogenic and organic causes of abnormal desire, arousal, orgasm, and sexual pain disorders based on physiologic and psychologic pathophysiology and includes a personal distress criterion for most diagnoses. Although the APA recognizes that female SD for many women is physically disconcerting [1], the DSM classifications are specifically limited to psychiatric disorders and are not intended to be used for evaluating or differentiating physical aspects of SD [1]. Moreover, sexual disorders, such as dyspareunia and vaginism, are typically diagnosed independent of etiology, which may be largely or entirely physical in some instances.

Dyspareunia and vaginism are both in the spectrum of painful intercourse, the difference being a matter of severity [18]. The DSM V classification stresses that they are penetration disorders in that any form of vaginal penetration, such as with tampons, finger, vaginal dilators, gynecological examinations, and intercourse, are painful (dysparenuria and vaginism) or impossible (vaginism). These conditions are still often underdiagnosed and therefore inadequately treated despite affecting millions of women worldwide [19]. Moreover, psychiatrists and psychologists find it difficult to differentiate between dyspareunia and vaginism [1]. The prevalence of dyspareunia and vaginism is about 8–16%, mostly involving diagnoses of vulvar vestibulitis or vulvodynia [20]. Other literature estimates the prevalence of female SD resulting from chronic pelvic and sexual pain to be 26% (range 7–58%) [21]. Provoked vestibulodynia (PVD) is another common subtype of vulvodynia, affecting ~12% of women [22]. Vaginism is reported to affect up to 21% of women <30 years [23], with an cumulative incidence of 10% of women unable to have sexual intercourse because of pain.

Gynecologists and related medical professionals frequently focus on assessment, evaluation, and treatment of peripheral manifestations and location of CPPS. Central sensitization and myofascial dysfunction are overlooked in many cases, probably due to lack of training in the assessment of myofascial dysfunction [7]. Little more than one decade ago physiotherapy for pelvic pain and female SD was almost nonexistent, with few studies reporting on this subject. The well-known psychiatrist Rosemary Basson [24] categorized the diagnosis and definition of major categories of women’s SD and their management. Vaginism was defined as persisting or recurrent difficulties in allowing vaginal entry of any object, despite the woman’s expressed wish to do so. The behavioral component was mentioned as the main source for management, without any reference to physiotherapy. For dyspareunia, the authors of that article suggested treatment with cyclic antidepressants, with or without pelvic muscle physiotherapy, without any further specification [24]. Important work by this group introduced the concept of a circular response cycle in women, termed the female sexual response cycle [25]. Their research stated that: next to sufficient sexual stimuli and motivation, the women’s state of mind, thought processes, beliefs, and emotions, might be the most important part of the sexual response cycle; the woman most likely would become more aroused and would desire sex more when in safe and secure surroundings and circumstances; and mentioned that anxiety or distraction because of pain or discomfort may limit the woman to be open or agree to having sex, which could hinder sexual arousal and desire. Therefore, in this cycle, there are not only psychological factors but physiological and physical factors that also play an important role. The question, then, is: Why did they relegate physiotherapy to a minor role in this cycle?"

There is a new, evolving model in which each individual is seen as a social–psychosomatic entity with an intricate and variable interaction of physical factors (genetic, phenotypically, biochemical, etc), psychological factors (mood, personality, behavior, etc.), and social factors (cultural, familial, socioeconomic, medical, etc.) [26]. This recent biopsychosocial model applies to disciplines ranging from medicine to psychology to sociology; its novelty, acceptance, and prevalence vary across disciplines and cultures [26, 27]. However, this model is very useful for understanding and evaluating the complexity of pelvic pain and female SD, which are often multifactorial, requiring a multidisciplinary assessment and treatment approach.

So far, there has been a tendency to view pelvic pain, dyspareunia, and vaginism as psychological distress resulting in a form of somatic or physical symptoms, for which medication or surgical intervention is necessary. The medical doctor, psychiatrist, or psychologist often considers these symptoms to be initiated and/or perpetuated by emotional responses, such as anxiety and depression. With this in mind, and to answer the question of where physiotherapy fits in the sex response cycle, it is important to understand the rationale of and relationship between the medical International Classification of Diseases (ICD)-10, the APA’s DSM V, and International Classification of Functions (ICF) guidelines for pelvic physiotherapy [28].

Whereas medical doctors use the ICD-10 to code the diagnosis of pelvic pain and SD, and psychiatrists, psychologists, and sexologists use the DSM V to classify them, physiotherapists use the ICF (Table 1) [28].

**Table 1:** Definitions of the International Classification of Functioning Terms: impairment, disability, and restriction in participation

Terminology	Definition
Impairment	Loss or abnormality of psychological, physiological, or anatomical structure or function at organ level
Disability	Restriction or loss of ability of a person to perform functions/activities in a normal manner
Restriction in participation	Disadvantage due to impairment or disability that limits or prevents fulfillment of a normal role (depends on age, sex, sociocultural factors) for the person

Using the ICF, the physiotherapist tries to influence the consequences of pelvic pain and SD on three different levels: organ (impairment/disorder level, e.g., intravaginal pain at penetration), personal (disability level, e.g., inability to have intercourse), and social–societal (restriction of participation, e.g., avoidance of sexual relationship = behavioral consequence). Where as the DSM V acts mainly on the psychological aspects of the personal and social–societal levels with the focus on (changing inadequate) behavior, the ICF incorporates organ levels considering local physical disorders and impairments [28].

**Fig. 1:**
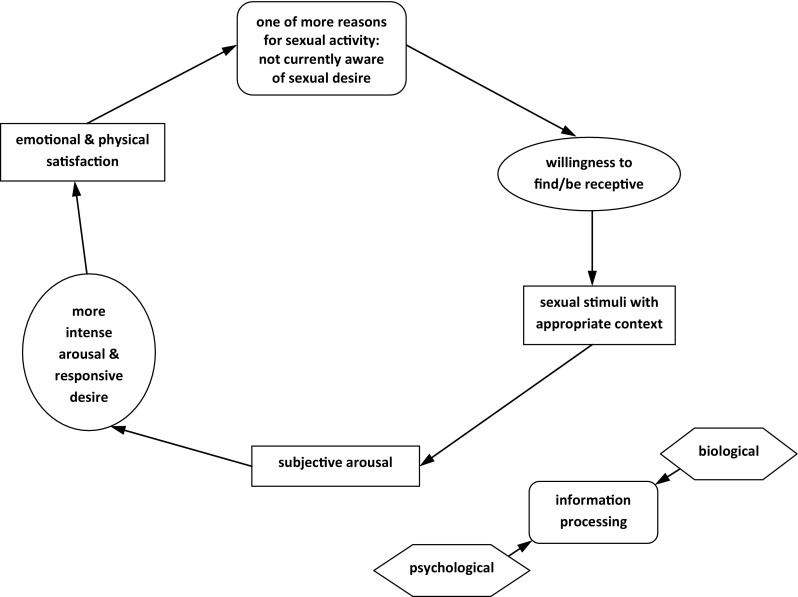
The female sexual response cycle (adapted from [25])

In Basson’s sex response cycle (Fig. 1), elements such as psychological and biological processing, arousal and responsive sexual desire, multiple reasons and incentives for instigating or agreeing to sex, and motivation require both adequate psychological and physical responses. Local pain or MTrP, overactive PFM, central sensitization-related hyperalgesia, and anxiety may hinder the state of mind around sex and/or sexual activity. In this setting, pelvic physiotherapy may be an important treatment co-interevention with psychological counseling.

As an example of this interplay, the cause of pelvic pain and dyspareunia might be due to an injury to the PFM, connective tissue, or fascia as a result of a birth trauma, sexual or physical abuse, or episiotomy during vaginal delivery. This can lead to shortened and weak PFM, with MTrPs and restriction of connective tissue resulting in chronic pelvic pain and dyspareunia. Ongoing and unresolved local injuries may lead to spinal cord central sensitization, and dorsal-root reflexes at the spinal cord level lead to referred symptoms of frequency, urgency, and nocturia, and the eventual development of noninfectious cystitis. This, in turn, provokes more pain, urgency, frequency, and nocturia, resulting in further contraction/tension of PFM, with further shortening and restriction of connective tissue. [13, 14]. To date, it was the rare clinician who, when assessing cystitis, would take into consideration that its cause is related to a latent injury of the pelvic floor, with consequential central sensitization and dorsal-root reflexes. Most likely, the clinician would continue the diagnostic process with invasive techniques or prescription of medication, rather than referring the patient to pelvic physiotherapy. Women with pelvic pain and SD constitute a group of patients with significant morbidity. They do not merely experience pain; the pain causes difficulty in walking, maintaining a normal life, work ability, and social interactions.

As mentioned, pelvic pain and female SD are often related to PFM dysfunction, such as overactive or underactive pelvic floor or coordination disorders. Stress as the central factor provokes a vicious cycle, with pain leading to muscle tension, pressure, nerve entrapment, and reduced circulation, which results in muscle shortening, which leads to restricted movement, creation of MTrPs, and further pain as a consequence [29]. The cause of stress and/or pain is not a simple or single problem but is complex and multiple. By the time women with CPP are diagnosed with PFM dysfunction, they already have undergone many unsuccessful therapeutic trials that often provided no adequate relief. According to the interviewed key-opinion leaders, for such women, self-empowerment is essential, and the role of physiotherapy is critical. It is not just the pelvic floor but the global impact of pain on the body. Many patients not only have pelvic pain but many other musculoskeletal manifestations. The role of physiotherapy is to get to the heart of the matter by starting with the basics: helping the patient to stand, sit, and walk differently. In doing so, the musculoskeletal impact of decreased strain and improved comfort is the first result many patients notice. Then, the physiotherapist can move on to working specifically on the pelvic floor. 

A multidisciplinary protocol, with a central role being physiotherapy, was developed at the University College Hospital, London, UK, with reported high levels of clinical efficacy and patient satisfaction (personal communication, Dr. Sohier Elneil, 2017). Physiotherapists need to be specifically trained and skilled in how to help patients avoid pain using cognitive and/or behavioral models, how to actively listen, and then how to display empathy for the emotional component of their patients’ disease [29].

In case of a referral to a pelvic physiotherapist, an accurate medical diagnosis is very important to determine the severity and impact of the disorder and to estimate success or failure of pelvic floor physiotherapy. In many cases, the presumed medical diagnosis (indication’) lacks accuracy, and physiotherapists are thus confronted with heterogeneity and unclear grade of severity, which may limit success or even result in failure. That many women with dyspareunia or vaginism have high levels of anxiety in response to facing a physical examination of external and internal pelvic structures leads to conditions being underdiagnosed [30]. Relevant scientific studies show that medical doctors did not specify pain location in 93%, duration in 44%, pathology in 74%, comorbidities in 95%, and additional inclusion/exclusion criteria in 65% of cases referred to physiotherapy [2, 31]. In a review by Kavvadias et al., which included 69 articles, the site of pain was specified in only 45% of studies, and only 20% of medical doctors performed a digital examination of pelvic MTrPs for diagnosis [32]. Thus, because of lack of sufficient relevant medical information, referral data, and test results, focused physiotherapy was difficult to administer adequately.

There is a recent tendency to more frequently involve physiotherapists in the multidisciplinary assessment and treatment of female SD and pain management. Multidisciplinary guidelines [2] and protocols (University College Hospital, 2017) are now available, and the Pain Clinic of the University Medical Center Groningen (UMCG), Groningen, The Netherlands, has developed the Pain Medicine Management Model. Whereas, in the past, the patient was assessed by one medical doctor and followed immediately by a treatment, assessment is now based on the patient’s complaint, and a multidisciplinary approach to both evaluation and treatment is central to the new model. Through a multidisciplinary assessment, including a thorough history and physical exam, with additional testing/examination, as indicated, the team develops a comprehensive diagnosis that includes presumed pathophysiology of the (dominant) pain mechanism. The UMCG multidisciplinary pain center team consists of a medical team (urologist, gynecologist, surgeon), a psychologist (psychologist/psychiatrist/sexologist), and a physiotherapist. This team assess predominant nociceptive, neuropathic, nonneuropathic, somatic, visceral, and referred neuropathy, evaluating for the presence of peripheral and central sensitization and taking into account any provoking and perpetuating biopsychosocial factors. The assessment takes 1 h in each discipline. The patient can then be classified and the plan of care tailored accordingly [33].

In the following paragraphs, physiotherapeutic assesment and treatment are described and scientific evidence discussed.

### Physiotherapeutic assessment and treatment

Cacchioni et al. examined and reported in detail sexual therapy for women involving body work: i.e., touch [34]. Women seeking advice for sexual problems are assessed and treated using close scrutiny, measurement, and response to touch of the genital area by health-care providers, including a medical doctor and pelvic physiotherapist [34]. Treatment may also involve instructing the woman in genital self-touch. A useful tool for managing CPP and female SD might be the so-called five-step ALLOW algorithm introduced by Sadovsky and Mulhall [35]. Only once the current step has been fulfilled satisfactorily for both the patient and the physiotherapist is the next step initiated:

Step 1: A: ask the patient whether you can proceed, then
Step 2: L: legitimize each part of the body work in such a way that the patient feels completely in control
Step 3: L: limitations, meaning that before and during body work the physiotherapist is, at all times, aware of his/her own competence and skill level and the patient’s emotions and feelings, referring, if necessary, to another professional
Step 4: O: be open for further discussion and evaluation with the patient and, if necessary, other competent colleagues or disciplines of the multidisciplinary team
Step 5: W: work to develop a treatment plan with the patient and other disciplines

### Physical exam

Before beginning the physical exam, the physiotherapist informs the patient about the nature of the procedure, helps the patient feel comfortable, and sets clear boundaries [34]. The physiotherapist also explains the difference between the objectives and execution of the physical exam to be performed by their physician and physiotherapist. The physiotherapist attends to women’s immediate complaints of sexual discomfort or displeasure, using body work to encourage them to feel more in control during the procedures and sexual activities. Patients have described the body work as therapeutic and empowering. Assessment and treatment modalities use visualization and hands-on techniques that stimulate patient reconnection with their bodies, rather than simply expressing a sensation of objective feelings. These strategies are often highly valued by patients because of the careful and gradual approach, which encourages women to be active participants in the overall process [34].

After a general inspection of posture and stability of spine and pelvis, the physical exam begins with inspection of the abdominal wall and observation of the patient’s breath while in the supine position. The perineal region is then examined, observing skin (color, temperature), scars, irregularities, moisture, etc. Next, a neuromuscular exam, assessing dermatomes, myotomes, searching for MTrPs, allodynia (skin-rolling test, pinch-and-roll technique), hyperalgesia (Wartenberg pinwheel), nerve entrapment, and pain points is performed. Muscle activity (tone) of lower back, hip, leg, and abdominal muscles is examined using palpation. Pelvic floor muscle activity (tone), spasms, and relaxation is assessed using internal palpation and/or biofeedback [7, 36].

More detailed information can be found elsewhere [37].

### Treatment

Information about the patient’s underlying health problem and education are always the starting point of treatment. Education includes explaining CPP pathophysiology and female SD, involvement of PFM, healthy vulvovaginal and sexual behaviors, factors influencing pain intensity, relaxation techniques, sexual function, and recovery of nonpainful sexual activities [38]. Physiotherapist-assisted stretching of the muscles of the back, lower extremities, and abdomen, in addition to nerve gliding to facilitate movement in restricted nerves, is important [30]. Stretching and strengthening techniques are then introduced to address muscle weakness, allowing for balance and stability.

As central sensitization and myofascial involvement may contribute to CPP and associated SD, physiotherapists use strategies that address treatment of MTrPs and pain regions, especially those that have been clinically tested and enhanced by scientific studies. Myofascial release involves physiotherapy and manual therapy modalities, including deep-pressure massage, stretching, joint mobilization, foam rollers [39], and other triggerpoint release techniques, such as vibration, transversal or flat palpation [39, 40] and dry needling [41]. At each session, ~30 min is dedicated to these manual techniques to increase flexibility, decrease TrP-related pain and tension, and increase balance and stability. Other pain management strategies, including general and specific respiratory and relaxation exercises, aim to enhance patient’s self-management and self-empowerment skills [11, 42]. Aredo et al. reported: “this dual approach addresses physiological and psychological components of chronic myofascial pain, alleviates MTrP-related pain, and furnishes patients with coping strategies to redirect their focus during a painful episode” [7].

Other frequently used treatment modalities are pain management programs to promote behavior change [43], PFM training [44, 45], biofeedback, electrical stimulation [46], and balloons and pelots for dilitation of vaginal tissues [47]. Goldstein et al. described a program of PFM training for vulvodynia [30] involving pelvic and core mobilization and stabilization techniques; connective tissue, visceral, and neural mobilization; and internal and external MTrP° release. Biofeedback and electrical stimulation assisted in decreasing tender points and tissue restrictions. The aim was to restore the proper length of the PFM and tissues, decreasing neural tension and dyspareunia. Vaginal dilators are recommended to normalize muscle tone, desensitize hypersensitive areas of vulva and vagina, and restore sexual function [48]. The daily home maintenance program involves relaxation and respiratory exercises, PFM training, stretching techniques, and the use of vaginal dilatators, if indicated [38].

### Scientific evidence for pelvic physiotherapy

Recently, some qualitative studies have been published on the effects of pelvic physiotherapy for CPP and female SD. Weiss et al. reported that regular in-clinic and at-home PFM training augments the support function of the pelvic floor, increases blood flow, and stimulates PFM proprioception, contributing to more intense orgasm [49]. In a review on chronic pelvic floor dysfunction, Hartmann et al. concluded that referral to a pelvic physiotherapist should occur routinely as part of the multidisciplinary approach for all women who present with any type of vulvovaginal pain [5]. Research indicates that pelvic physiotherapy is safe and effective and can dramatically improve symptoms related to CPP and chronic PFD. Pelvic physiotherapy stimulates self-empowerment of women and supports recovery of function they may have lost due to pain and dysfunction. Sadownik et al. [50], in a qualitative retrospective study; Brotto et al. [51], in a longitudinal prospective study; Goldstein et al. [30], in a report of the expert committee of the Fourth International Consultation on Sexual Medicine; and Goldfinger et al. [52], in an RCT; emphasize the efficacy of pelvic physiotherapy as part of the multidisciplinary approach for CPP and SD. Goldstein et al. stated that physiotherapist-assisted stretching of all muscles related to the pelvis, abdomen, low back and upper legs, in addition to nerve gliding to facilitate movement in restricted nerves, is necessary to improve CPP and SD [30]. The authors reported that stretching exercises and strength training restored balance and stability, proper PFM and fascia tissue length, and decreased neural tension and dyspareunia. In a RCT Goldfinger et al. investigated effects on provoked vestibulodynia by comparing cognitive behavioral therapy and multimodal physiotherapy [52]. The physiotherapy protocol combined education, PFM exercises, manual techniques, surface electromyographic biofeedback, progressive vaginal penetration exercises through the use of four silicone vaginal dilators of varied diameter, stretching of hip muscles, deep breathing, global body relaxation exercises, and pain management techniques. They concluded that both interventions are effective treatment options for women with provoked vestibulodynia. Sadownik et al. stated that behavioral change stimulated by physiotherapy that enhances the patient’s bodily experience is an important aspect in improving self-efficacy and decreasing the experience of overly negative cognitions [50].

One RCT found that vaginal electrical stimulation improved the sexual experience of women with PFD who scored low on the Female Sexual Function Index (FSFI) [53]. A longitudal prospective study showed that transcutaneous electrical nerve stimulation (TENS) was feasible and beneficial for treatment-resistant provoked vestibulodynia [54]. In an RCT of women with pelvic and sexual pain, Zoorob et al. concluded that pelvic physiotherapy improves sex life and decreases pain in an equivalent response to injections [55]. An RCT comparing the effect of physiotherapy with surgery resulted in similar outcomes [56].

## Conclusions

CPP and female SD are prevalent and multifactorial issues that threaten women’s quality of life. As part of the multidisciplinary team, and because of its holistic and whole-body approach, pelvic physiotherapy can contribute significantly to assessing and treating such women, and clinical and scientific research indicate its efficacy and safety. The role of pelvic physiotherapy for these patients remains a relatively untapped resource. Further high-quality RCTs are warranted for several physiotherapeutic modalities and protocols and to determine their long-term effects in the integrated treatment plan of women with CPP and SD.
